# Genetic Dissection of Maize Embryonic Callus Regenerative Capacity Using Multi-Locus Genome-Wide Association Studies

**DOI:** 10.3389/fpls.2018.00561

**Published:** 2018-04-26

**Authors:** Langlang Ma, Min Liu, Yuanyuan Yan, Chunyan Qing, Xiaoling Zhang, Yanling Zhang, Yun Long, Lei Wang, Lang Pan, Chaoying Zou, Zhaoling Li, Yanli Wang, Huanwei Peng, Guangtang Pan, Zhou Jiang, Yaou Shen

**Affiliations:** ^1^Key Laboratory of Biology and Genetic Improvement of Maize in Southwest Region, Maize Research Institute, Sichuan Agricultural University, Chengdu, China; ^2^Institute of Animal Nutrition, Sichuan Agricultural University, Chengdu, China

**Keywords:** maize, embryonic callus, regenerative capacity, multi-locus GWAS, candidate gene

## Abstract

The regenerative capacity of the embryonic callus, a complex quantitative trait, is one of the main limiting factors for maize transformation. This trait was decomposed into five traits, namely, green callus rate (GCR), callus differentiating rate (CDR), callus plantlet number (CPN), callus rooting rate (CRR), and callus browning rate (CBR). To dissect the genetic foundation of maize transformation, in this study multi-locus genome-wide association studies (GWAS) for the five traits were performed in a population of 144 inbred lines genotyped with 43,427 SNPs. Using the phenotypic values in three environments and best linear unbiased prediction (BLUP) values, as a result, a total of 127, 56, 160, and 130 significant quantitative trait nucleotides (QTNs) were identified by mrMLM, FASTmrEMMA, ISIS EM-BLASSO, and pLARmEB, respectively. Of these QTNs, 63 QTNs were commonly detected, including 15 across multiple environments and 58 across multiple methods. Allele distribution analysis showed that the proportion of superior alleles for 36 QTNs was <50% in 31 elite inbred lines. Meanwhile, these superior alleles had obviously additive effect on the regenerative capacity. This indicates that the regenerative capacity-related traits can be improved by proper integration of the superior alleles using marker-assisted selection. Moreover, a total of 40 candidate genes were found based on these common QTNs. Some annotated genes were previously reported to relate with auxin transport, cell fate, seed germination, or embryo development, especially, GRMZM2G108933 (*WOX2*) was found to promote maize transgenic embryonic callus regeneration. These identified candidate genes will contribute to a further understanding of the genetic foundation of maize embryonic callus regeneration.

## Introduction

As one of the main crops for animals and humans, maize (*Zea mays* L.) is an important target for genetic manipulation (Zhang et al., 2014; Li et al., 2016). However, during maize transformation, difficulty in embryonic callus induction and regeneration, which occurs in most elite lines, presents a major bottleneck (Shen et al., 2012, 2013; Ge et al., 2016). Previous studies have suggested that both genotypes and exogenous hormones affect embryonic callus induction from maize immature embryos, such as abscisic acid (ABA), indole acetic acid (IAA), and gibberellic acid (GA3) widely considered to play important roles in callus formation (Jiménez and Bangerth, 2001; Ge et al., 2016). Genetic research has suggested that embryonic callus induction is controlled by nuclear genes in maize (Schlappi and Hohn, 1992). Furthermore, eight quantitative trait loci (QTL) and three epistatic interactions were found to control type I callus formation in a maize recombinant inbred line (RIL) population (Krakowsky et al., 2006). In previous studies, some transcription factors and microRNAs in hormone signal transduction pathways were found to regulate the process of embryonic callus induction (Shen et al., 2013; Ge et al., 2016). To date, research exploring callus regenerative capacity has mainly focused on *Arabidops*is, rice, wheat, maize, and other plants. In *Arabidopsis, PLT* genes (PLETHORA) were proved to modulate the regenerative capacity by a two-step mechanism (Kareem et al., 2015). First, *PLT3, PLT5*, and *PLT7* activated the expression of root stem cell regulators *PLT1* and *PLT2* to establish pluripotency and form shoot progenitors. Then, *PLT3, PLT5*, and *PLT7* up-regulated the expression of shoot-promoting factor Cup-shaped cotyledon1 (*CUC1*) and Cup-shaped cotyledon2 (*CUC2*) to complete the shoot regeneration process. Inhibitor of cyclin-dependent kinase (*ICK*), a cyclin-dependent kinase (*CDK*) inhibitor, has been shown to enhance the regenerative capacity of *Arabidopsis* embryonic callus (Cheng et al., 2015). Moreover, WUSCHEL-related homeobox 5 (*WOX5*) expression in the quiescent center (QC) is considered as a marker of the root stem cell niche in *Arabidopsis* (Sarkar et al., 2007). In addition, as an AP2/ERF transcription factor, wound induced dedifferentiation1 (WIND1) promoted the *Arabidopsis* shoot regeneration by up-regulating the expression of enhancer of shoot regeenration1 *(ESR1)* gene which encoded another AP2/ERF transcription factor (Iwase et al., 2015, 2017). For wheat, genes controlling green shoot re-differentiation were mapped to chromosomal sites 3A, 5B, 2D, and 1B (Szakács et al., 1988). Additionally, QTL mapping showed that two QTLs on chromosomes 1 and 9 control green shoot re-differentiation in rice, with the latter considered to be a major locus (Ping et al., 1998). Nishimura et al. (2005) observed a main QTL encoding ferredoxin-nitrite reductase (NiR) which is responsible for regenerate ability in rice. Recently, WUSCHEL-related homeobox 2 (*WOX2*) and Baby Boom (*BBM*) genes were introduced into maize by genetic transformation, which resulted in the increased rate of resistant seedlings from transformed immature embryos (Lowe et al., 2016). So far, the genetic basis of plant regeneration has not been well understood especially for maize, in which few functional genes have been revealed to directly control embryonic callus regeneration. Therefore, more systematic studies are required to reveal the genetic basis of maize embryonic callus regenerative capacity.

Genome-wide association analysis (GWAS) is a useful tool in the dissection of complex traits (Abdel-Ghani et al., 2013; Pace et al., 2015). Using mixed linear model (MLM) and general linear model (GLM), 4 and 263 significant SNPs were found to be associated with root architecture traits at maize seedling stage, respectively. More specifically, GRMZM2G153722, which is located on chromosome 4, was found to contain nine significant SNPs that are likely expressed in the roots and shoots (Pace et al., 2015). When using GWAS, several genes that modulate maize leaf architecture were identified in a nested association mapping (NAM) population (Tian et al., 2011). GWAS also aided in the identification of 74 candidate genes associated with maize oil biosynthesis (Li et al., 2013). Furthermore, another study identified a total of 51 SNPs significantly associated with maize leaf blight by adopting a NAM population, with most of the candidate genes reported in previous studies as relating to plant disease resistance (Kump et al., 2011). To our knowledge, there is no study that has utilized GWAS when detecting the embryonic callus regenerative capacity until now.

In this study, four multi-locus GWAS approaches were used to dissect the genetic foundations for the five regenerative capacity-related traits in a natural population containing rich genetic information across multiple environments. Our objectives were: (i) to understand the significance of genotype, environment, and genotype × environment on traits relating to regenerative capacity; (ii) to identify significant quantitative trait nucleotides (QTNs) and candidate genes that modulate the five traits and resolve the genetic basis of maize embryonic callus regenerative capacity; and (iii) to analyze and compare the detection powers of different methods and identify the optimal multi-locus GWAS approach. To our knowledge, this is the first comprehensive study aimed at understanding the genetic basis of maize embryonic callus regenerative capacity using multi-locus GWAS approaches.

## Materials and methods

### Plant materials and phenotypic data analysis

**Figure 1:**
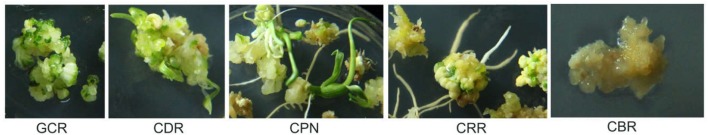
Features of the five traits. The traits include CBR (callus browning rate), CDR (callus differentiating rate), CPN (callus plantlet number), CRR (callus rooting rate), and GCR (green callus rate).

In a previous study, we examined the embryonic callus induction rate in immature embryos from a natural maize population of 362 inbred lines, with 144 of the lines exhibiting efficient induction (Table S1) and thus they were used to detect regenerative capacity. The details of planting and culturing processes were described by Zhang et al. (2017b). Herein, five regeneration ability-related traits, namely, embryonic green callus rate (GCR), callus differentiating rate (CDR), callus plantlet number (CPN), callus rooting rate (CRR), and callus browning rate (CBR), were examined (the features of the five traits were shown in Figure 1). The data were transformed as previously described with the GCR, CDR, CRR, and CBR values calculated by ${\text{sin}}^{-1}\sqrt{\text{p}}$ and the CPN value calculated by $\sqrt{\text{p}+1}$, with p being the initial value (Zhang et al., 2017b). The analysis of variance (ANOVA), phenotypic correlation, BLUP values and broad-sense heritability ($H_{B}^{2}$) were all completed in our previous study (Zhang et al., 2017b).

### Genotypic data analysis

Genomic DNA was extracted from mixed leaf tissues from eight plants per line using the CTAB method (Zhang et al., 2016). All of the accessions were genotyped using the Illumina MaizeSNP50 BeadChip containing 56,110 SNPs (http://support.illumina.com/array/array_kits/maizesnp50_dna_analysis_kit/downloads.html). A total of 43,427 SNPs across 10 chromosomes remained after quality filtering (Figure S1), with SNPs having a missing rate >20%, heterozygosity >20%, and minor allele frequency (MAF) < 0.05 deleted. These 43,427 SNPs were subsequently used for calculating the population structure and kinship and to perform GWAS.

### Population structure, linkage disequilibrium, and multi-locus association studies

STRUCTURE 2.3.4 was used to estimate subgroup numbers within the population structure (Q matrix) (Evanno et al., 2005). Among the 43,427 SNPs, 5,000 high quality SNPs with a rare allele frequency (RAF) >20% were randomly selected for the estimating panel. Based on the subgrouping results, the obtained evaluated data were used for further analysis.

TASSEL 4.0 was utilized to analyze linkage disequilibrium (LD) (Bradbury et al., 2007), with the LD decay calculated by plotting *r*^2^ onto the genetic distance in base pairs with a cutoff of *r*^2^ = 0.2. The LD decay was calculated using only markers that remained after quality filtering. Additionally, the Loiselle kinship coefficients between inbred lines in a panel (K matrix) were calculated using SpAGeDi software (Hardy and Vekemans, 2002).

In this study, four multi-locus GWAS approaches were used to detect significant QTNs for five embryonic callus regenerative capacity-related traits (mrMLM v2.1, https://cran.r-project.org/web/packages/mrMLM/index.html), including mrMLM (Wang et al., 2016), FASTmrEMMA (Wen et al., 2017), ISIS EM-BLASSO (Tamba et al., 2017), and pLARmEB (Zhang et al., 2017a). Owing to the fact that these multi-locus methods were more powerful and accurate than the single-locus MLM methods in their simulation experiments, thus we adopted these multi-locus methods in this study. Moreover, Q- and K-matrices were applied to correct the population structure and Loiselle kinship coefficients that were calculated between inbred lines. The setting parameters for these methods were as follows: (i) mrMLM, critical *P*-value of 0.01 in rMLM and critical LOD score of 3.0 in mrMLM (Wang et al., 2016); (ii) FASTmrEMMA, critical *P*-value of 0.005 in first step of FASTmrEMMA and critical LOD score of 3.0 in the last step of FASTmrEMMA (Wen et al., 2017); (iii) ISIS EM-BLASSO, critical *P*-value of 0.0002 in ISIS EM-BLASSO (Tamba et al., 2017); and (iv) pLARmEB, critical LOD score of 3.0 in pLARmEB and the number of potentially associated variables for each chromosome: 143 (“144–1”) (Zhang et al., 2017a).

### Superior allele analysis and annotation of candidate genes

For QTNs (RefGen_v2) that were detected consistently in multiple environments or methods, a superior genotype was determined based on the effect value of each significant QTN. For each QTN, the superior allele percentage in these elite inbred lines was equal to number of lines containing superior alleles divided by the total line number. For each line, the proportion of superior alleles in these QTNs was calculated as superior allele number divided by total QTN number. A heat map visualizing the percentage of superior alleles was obtained in the R (heatmap package) program (Mellbye and Schuster, 2014).

Herein, the QTNs which locate in gene regions were used to identify the candidate genes. Furthermore, the corresponding candidate genes of the consistent QTNs that were stably expressed in multi-environment or multi-method were annotated by performing a GENE search on the NCBI website (RefGen_v2) (https://www.ncbi.nlm.nih.gov/).

### Real-time PCR for candidate genes

Four candidate genes GRMZM2G108933 (*WOX*2), GRMZM2G066749, GRMZM2G163761, and GRMZM2G371033 were randomly selected for identification of expression levels at different regeneration stages (0 d, 3 d, 6 d, and 9 d) by quantitative real-time PCR analysis (qPCR, ABI 7500 real-time PCR System, Torrance, CA, USA). Firstly, RNA samples were extracted using TRIZOL reagent (Invitrogen, Beijing, China) and RNase-free DNase (Takara, Beijing, China). Then, cDNA was obtained by PrimeScript RT Reagent Kit With gDNA Eraser (TaKaRa, Beijing, China). Moreover, the primers were designed using the software Primer Premier 5.0. The detailed PCR amplification programmes were described as Shen et al. (2012), and the 2^−ΔΔCt^ method was used for calculating the expression levels (Schefe et al., 2006). Here, *Actin* 1 (GRMZM2G126010) was used as the reference gene.

## Results

### Phenotype for regenerative capacity-related traits

The phenotypes for CBR, CDR, CPN, CRR, and GCR have been described by Zhang et al. (2017b), readers are encouraged to refer to the original study (Zhang et al., 2017b). The results were briefly described here. The average values for the above five traits across three environments were 37.70, 17.30, 1.28, 11.50, and 43.16 with the standard deviations 26.99, 17.52, 0.51, 14.25 and 24.94, respectively. Additionally, the heritability ($h_{\text{B}}^{2}$) of the five traits ranged from 47.09 to 78.91%, suggesting that genetic effects play an important role in the formation of these traits. A significantly positive correlation was observed between CDR and CPN, while a significantly negative correlation was found between CBR and GCR (*P* = 0.01). The high correlation coefficient between the BLUP value and the phenotypic value in a single environment (>0.9) indicated the reliability of the phenotypic values for most of the traits (Figure S2; Zhang et al., 2017b).

### Linkage disequilibrium decay in the population

To obtain the average distance of LD decay, 43,427 SNPs were adopted. As shown in Figure S3, *r*^2^ decreased gradually with increased distance. However, the *r*^2^-value reached a plateau when it decreased to a certain level. The corresponding distance was considered as the average distance of LD decay in this population. Herein, the average LD decay distance was 220 kb (*r*^2^ = 0.2), which is consistent with a previous study (Zhang et al., 2016). Moreover, the distance was greater than the average distance between markers of 48 kb, thus indicating sufficient coverage.

### Population structure

**Figure 2:**
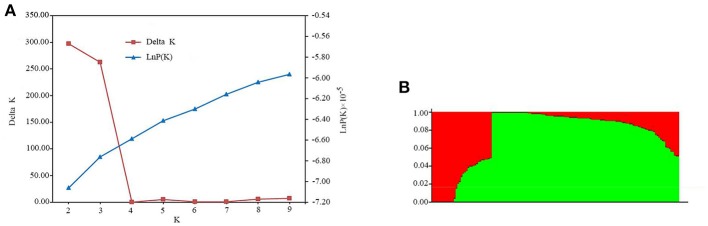
Population structure estimates based on 43,427 SNPs distributed across 10 chromosomes. **(A)** Plot of ln*P*(*D*), with Δ*K* calculated for *K* = 2–9. **(B)** Population structure estimates (*K* = 2), the areas of the two colors (green and red) illustrate the proportion of each subgroup.

A subset of 5,000 high quality SNPs were randomly chosen to define the subpopulations within the panel of 144 lines. Delta *K* (Δ*K*) was calculated using STRUCTURE 2.3.4 (Figure 2A; *K* = 2–9), with two subpopulations (selected *K* = 2) presented based on Δ*K*-values (Figure 2B). These two subgroups contained 109 (75.69%) and 35 (24.31%) lines (Table S1), respectively. The larger subpopulation included tropical, temperate, and mixed germplasms, while the other was composed of mostly temperate lines (Table S1).

### QTNs detected by multi-locus GWAS methods

**Figure 3:**
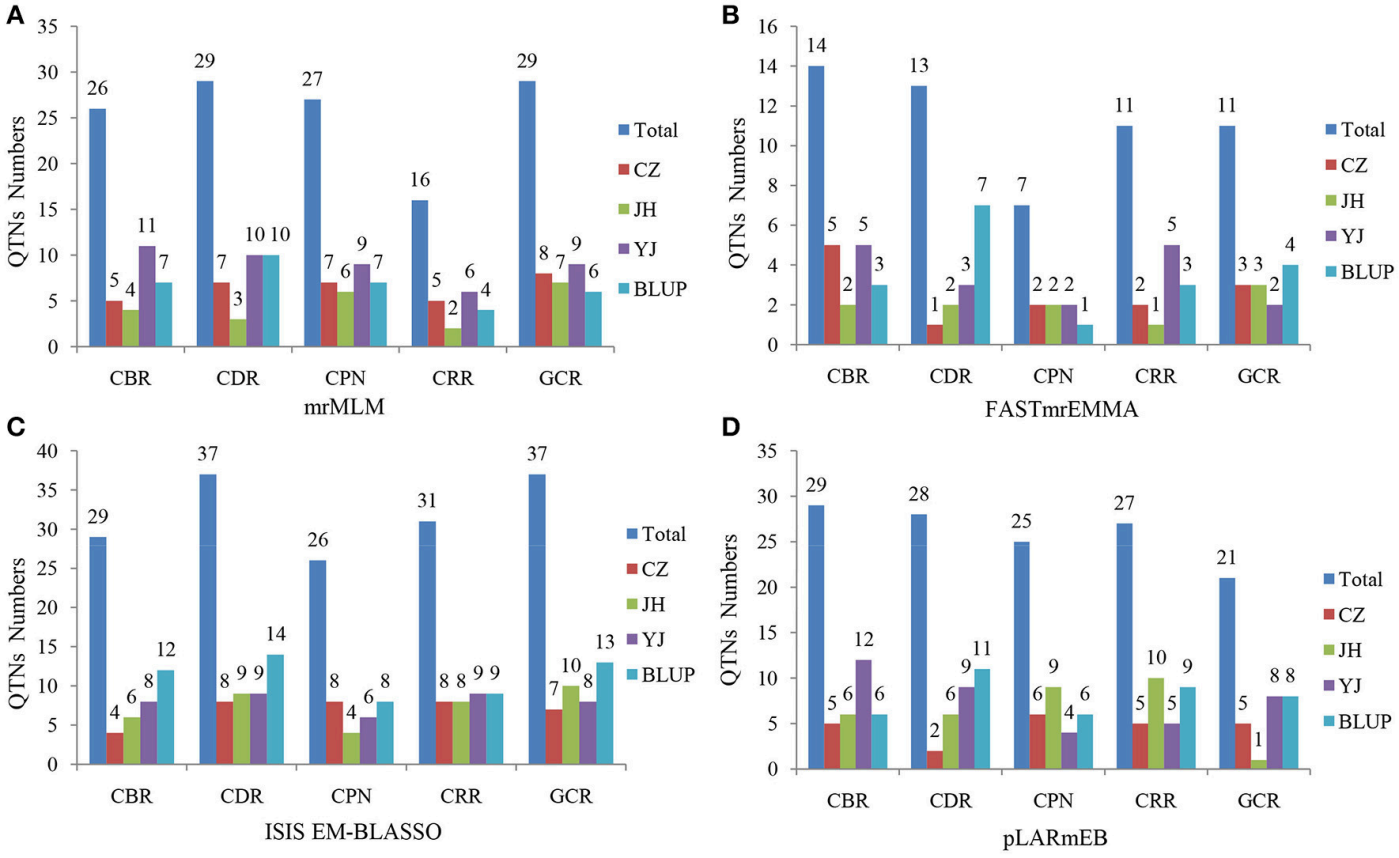
Number of detected QTNs for the five traits across three environments and BLUP model in four methods. The traits include CBR (callus browning rate), CDR (callus differentiating rate), CPN (callus plantlet number), CRR (callus rooting rate), and GCR (green callus rate). CZ, JH, and YJ denote the population planted in Chongzhou (2015), Jinghong (2014), and Yuanjiang (2015), respectively. The approaches utilized included **(A)** mrMLM, **(B)** FASTmrEMMA, **(C)** ISIS EM-BLASSO, and **(D)** pLARmEB.

**Figure 4:**
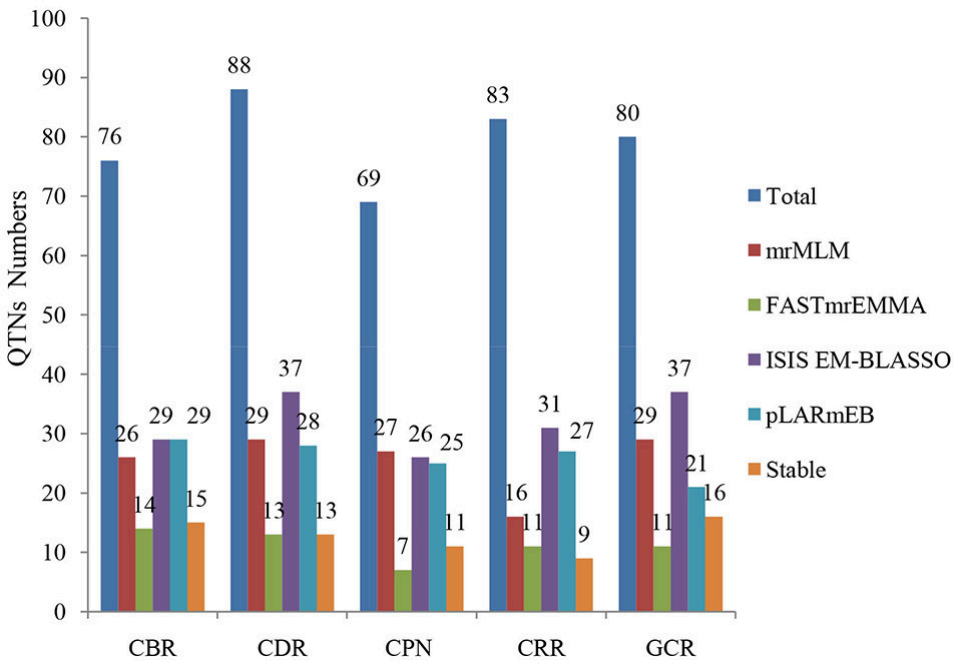
Comparison of the number of detected QTNs from the four methods. The four methods are mrMLM, FASTmrEMMA, ISIS EM-BLASSO, and pLARmEB. The traits include CBR (callus browning rate), CDR (callus differentiating rate), CPN (callus plantlet number), CRR (callus rooting rate), and GCR (green callus rate). Total: denotes the total QTN number for each trait and Stable: denotes the number of stably expressed QTNs across multiple methods for each trait.

Four multi-locus GWAS approaches were utilized in this study. A total of 127, 56, 160, and 130 significant QTNs were identified in mrMLM, FASTmrEMMA, ISIS EM-BLASSO, and pLARmEB, respectively, for five traits across three environments and the BLUP model (Figures 3, 4, Figure S4, Tables S2–S5). Among them, 26, 29, 27, 16, and 29 QTNs were identified for CBR, CDR, CPN, CRR, and GCR, respectively, in multi-location and BLUP model by mrMLM method (Figure 3A; Table S2). When using FASTmrEMMA, the number of QTNs detected for the five traits were 14, 13, 7, 11, and 11, respectively (Figure 3B; Table S3). The ISIS EM-BLASSO method also identified 29, 37, 26, 31, and 37 QTNs for the above five traits (Figure 3C; Table S4). Moreover, 29, 28, 25, 27, and 21 QTNs were identified for the above five traits, respectively, using the pLARmEB approach (Figure 3D; Table S5).

We further analyzed the common QTNs that were co-identified in at least two of the environments (or environments and BLUP model) using a certain multi-locus GWAS approach. A total of 15 common QTNs were identified by combination of these four methods (Table 1). Among them, six, two, eight, and three environment-stable QTNs were identified using mrMLM, FASTmrEMMA, ISIS EM-BLASSO, and pLARmEB method, respectively (Table 1). These common QTNs were separately located on chromosomes 1, 2, 3, 4, 5, 7, 8, and 9, with LOD values ranging from 3.06 to 9.40 (Table 1). The proportion of phenotypic variance explained (PVE) by each QTN ranged from 1.83 to 19.26% (Table 1). Furthermore, three, four, two, six, and four common QTNs were found significantly associated with CBR, CDR, CPN, CRR, and GCR, respectively (Table 1).

**Table 1:** Stably expressed QTNs for the five traits in each method across three environments and BLUP model.

**Method**	**Trait**	**Environment and BLUP**	**Marker**	**Chromosome**	**Marker position (bp)**	**QTN effect**	**LOD score**	***r*^2^ (%)^a^**
mrMLM	CBR	YJ, BLUP	PZE-109066380	9	109,317,272	−16.82, −8.67	5.61, 3.23	13.92, 15.08
	CDR	CZ, BLUP	SYN15872	8	161,523,427	−6.45, −2.16	5.18, 4.75	8.44, 5.45
	CPN	CZ, BLUP	PZE-101220149	1	271,749,865	−0.28, −0.11	8.26, 4.42	14.86, 8.27
		CZ, BLUP	SYN39155	3	2,446,145	0.12, 0.06	3.85, 3.06	5.86, 5.14
	CRR	CZ, BLUP	PZE-101160089	1	202,300,686	−14.92, −3.37	8.18, 8.28	16.38, 19.26
	GCR	YJ, BLUP	SYNGENTA13688	2	5,681,488	−7.20, −6.40	4.26, 4.08	7.55, 9.82
FASTmrEMMA	CBR	CZ, BLUP	SYNGENTA15901	7	5,038,808	21.95, 5.60	7.63, 3.74	13.19, 4.77
	GCR	CZ, BLUP	PZE-104024889	4	28,985,737	−14.27, −7.42	3.31, 3.66	6.48, 5.99
ISIS EM-BLASSO	CBR	CZ, BLUP	SYN8267	4	169,213,008	−10.11, −1.80	5.36, 3.14	10.05, 1.83
	CDR	CZ, BLUP	SYN32084	1	256,515,262	5.76, 2.51	6.35, 8.87	7.25, 6.09
		CZ, BLUP	SYN39155	3	2,446,145	4.47, 1.69	4.76, 4.59	4.68, 3.10
		CZ, JH	PZE-107024505	7	26,451,809	−5.72, −4.80	3.54, 4.09	5.14, 4.39
	CRR	CZ, BLUP	PZE-101160089	1	202,300,686	−8.84, −1.97	3.26, 5.38	6.23, 6.74
		CZ, BLUP	SYN35026	5	1,946,471	−3.60, −0.83	3.28, 4.72	3.07, 4.09
		YJ, BLUP	PZE-107005556	7	3,824,391	−4.20, −0.75	6.29, 3.90	7.74, 3.76
	GCR	CZ, JH	PZE-102138070	2	186,820,524	8.49, 8.27	6.33, 9.02	10.12, 12.43
pLARmEB	CRR	CZ, BLUP	SYN35026	5	1,946,471	−8.14, −1.35	7.37, 9.40	17.27, 6.53
		JH, BLUP	PZE-107005556	7	3,824,391	−4.03, −0.70	4.47, 3.43	4.80, 2.00
	GCR	YJ, BLUP	PZE-104068814	4	136,958,099	−4.17, −3.01	3.18, 3.82	2.69, 4.74

*Traits include CBR (callus browning rate), CDR (callus differentiating rate), CPN (callus plantlet number), CRR (callus rooting rate), and GCR (green callus rate)*.

*JH, CZ, and YJ denote the population planted in Jinghong (2014), Chongzhou (2015), and Yuanjiang (2015), respectively*.

*a r^2^ (%), phenotypic variation of traits explained by each QTN*.

When comparing the results across different methods, 58 QTNs were consistently identified by two or more methods (Table 2), and they were associated with the CBR (15), CDR (13), CPN (11), CRR (9), and GCR (16) traits (Figure 4; Table 2). Especially, three QTNs (SYNGENTA15901, SYN39155, and SYN32084) were found to be significantly associated with CBR, CDR, and CDR, respectively, in all the multi-locus methods (Table 2). Meanwhile, the average LOD-values and PVE ranges of the three QTNs for the CBR (5.69; 4.77–13.19%), CDR (4.68; 3.10–6.45%), and CDR (7.61; 2.65–7.25%) traits were also generated (Table 2).

**Table 2:** Stably expressed QTNs for the five traits among different multi-methods.

**Trait**	**Method (1, 2, 3, 4)^a^**	**Marker**	**Chromosome**	**Maker position (bp)**	**LOD score**	***r*^2^ (%)^b^**
CBR	1, 3, 4	PZE-102138070	2	186,820,524	5.68, 3.92, 6.73	9.48, 5.00, 11.56
	1, 4	PZE-108009888	8	10,298,512	5.78, 3.17	8.01, 4.60
	1, 3	PZE-110088629	10	138,832,523	3.85, 3.35	5.43, 2.65
	1, 3	PZE-103123331	3	181,066,730	3.29, 4.53	6.40, 3.58
	1, 2, 3	SYN6514	3	196,351,287	3.56, 4.05, 7.36	3.63, 6.32, 8.30
	1, 3, 4	SYN7221	2	6,200,684	3.75, 11.83, 5.94	7.76, 10.78, 6.22
	1, 2	PZE-109067144	9	110,459,334	3.27, 3.14	2.59, 4.92
	1, 2, 3, 4	SYNGENTA15901	7	5,038,808	3.69, 3.74	10.75,4.77 (13.19)
					(7.63),7.03, 5.84	12.35, 11.39
	1, 3	PZE-102109721	2	141,363,433	4.18, 5.46	3.64, 4.91
	2, 3	PZE-102151093	2	197,600,202	5.56, 6.37	10.24, 12.66
	2, 3, 4	PZE-101213720	1	264,163,677	4.65, 6.84, 4.20	6.88, 11.24, 6.73
	2, 3, 4	SYN8267	4	169,213,008	5.19, 3.14	8.43, 1.83
					(5.35),7.29	(10.05), 16.35
	2, 4	PZE-101152052	1	19,548,4495	4.16, 6.94	6.30, 4.76
	2, 4	PZE-108020924	8	19,855,121	3.75, 13.53	4.89, 9.52
	3, 4	PZE-104067972	4	134,998,323	4.90, 9.53	4.89, 7.06
CDR	1, 4	PZE-106032634	6	75,630,749	16.15, 4.43	18.87, 5.68
	1, 2, 3, 4	SYN39155	3	2,446,145	4.73, 5.07, 4.59	4.85, 6.45, 3.10
					(4.76), 4.05	(4.68), 4.21
	1, 2, 3, 4	SYN32084	1	256,515,262	4.04, 3.05, 8.87	5.89, 2.65, 6.09
					(6.35), 8.74	(7.25), 5.19
	1, 2, 3	PZE-101216827	1	267,908,158	3.48, 4.33, 6.19	4.77, 4.23, 3.25
	1, 2	SYN11739	9	9,965,031	4.92, 3.66	14.28, 8.27
	1, 3	SYN31996	6	163,506,361	3.13, 3.86	9.16, 3.58
	1, 3	PZE-108002411	8	2,512,300	16.25, 4.97	14.64, 6.03
	1, 4	PZE-101096007	1	94,367,481	4.86, 4.45	3.72, 5.66
	1, 3	PZE-102109640	2	141,173,773	7.67, 5.68	4.86, 5.53
	3, 4	PZE-104025174	4	29,335,471	9.52, 3.01	7.33, 0.89
	3, 4	PZE-106036875	6	84,672,851	4.31, 6.72	1.97, 4.40
	3, 4	PUT-163a-31909945-2005	6	110,706,817	4.29, 3.58	2.04, 4.30
	3, 4	SYN8144	10	142,358,869	4.21, 4.09	3.31, 5.42
CPN	1, 3, 4	PZE-106032634	6	75,630,749	7.88, 4.58, 4.56	14.66, 7.12, 0.62
	1, 3	PZE-101220149	1	271,749,865	4.42 (8.26), 4.96	8.27 (14.86), 6.35
	1, 3	SYN39155	3	2,446,145	3.06 (3.85), 4.20	5.14 (5.86), 3.04
	1, 3, 4	PZE-108105282	8	159,954,599	3.88, 6.55, 5.42	6.91, 5.57, 1.57
	1, 4	PZE-108057325	8	102,454,042	3.07, 6.36	5.32, 0.47
	1, 3	PZE-104066682	4	131,771,972	7.49, 3.31	17.57, 8.73
	1, 3	PZE-106043314	6	93,212,668	6.64, 3.46	9.61, 4.20
	1, 2, 3	PZE-102186765	2	230,884,488	9.11, 7.26, 6.50	12.45, 0.30, 9.41
	2, 3	PZE-109062403	9	104,884,301	6.36, 13.00	16.33, 14.58
	3, 4	PZE-103049772	3	54,177,469	6.93, 12.72	14.03, 12.98
	1, 3	SYN29447	5	213,294,101	3.29, 6.18	5.79, 6.17
CRR	2, 3	SYN18315	1	252,377,691	7.13, 7.27	12.37, 9.45
	2, 3	PZE-106008760	6	25,291,385	6.76, 4.44	13.33, 6.04
	2, 4	PZE-101085779	1	75,627,286	4.81, 7.65	10.52, 6.47
	2, 3	SYN28088	5	68,653,887	4.18, 6.13	6.74, 5.74
	2, 3	PZE-106000504	6	1,234,387	3.04, 4.17	4.13, 2.57
	2, 3, 4	PZE-107005556	7	3,824,391	4.13, 3.90 (6.29)	9.14, 3.76 (7.74)
					3.43(4.47)	2.00(4.80)
	3, 4	SYN35026	5	1,946,471	4.72 (3.28)	4.09 (3.07)
					9.40 (7.37)	6.53 (17.27)
	3, 4	PZE-105122012	5	179,270,149	5.07, 7.25	4.17, 4.70
	3, 4	SYN18708	1	21,466,619	5.18, 4.33	4.15, 3.11
GCR	1, 3	SYN32084	1	256,515,262	3.99, 4.08	6.10, 4.35
	1, 3	SYNGETA13688	2	5,681,488	4.08 (4.26), 4.96	9.82 (7.55), 5.25
	1, 3, 4	PZE-109121058	9	154,807,596	8.07, 7.71, 5.76	13.73, 11.26, 8.20
	1, 3, 4	PZE-103123331	3	181,066,730	3.42, 4.03, 7.25	6.67, 7.75, 5.46
	1, 3	PZE-108010908	8	11,504,308	4.61, 6.26	6.88, 6.58
	1, 4	SYN37974	2	10,782,867	3.25, 6.41	7.78, 10.77
	2, 3	PZE-103108199	3	169,053,554	3.28, 5.74	4.47, 6.96
	2, 3	PZE-104024889	4	28,985,737	3.66 (3.31), 4.93	5.99 (6.48), 5.90
	2, 4	PZE-104069507	4	138,153,696	5.14, 4.45	10.42, 14.05
	2, 4	PZE-101106628	1	110,914,630	4.86, 5.53	15.03, 9.59
	3, 4	SYN7221	2	6,200,684	4.70, 3.42	3.57, 3.82
	3, 4	PZE-109081358	9	129,514,761	5.86, 6.96	3.98, 9.62
	3, 4	PZE-101223466	1	274,722,612	9.02, 7.28	12.32, 8.94
	3, 4	PZE-102138070	2	186,820,524	6.33 (9.02), 6.42	10.12 (12.43), 9.73
	3, 4	SYN21743	9	1,347,687	7.10, 5.60	9.66, 7.20
	3, 4	PZE-108021239	8	20,231,393	4.19, 4.09	4.87, 5.42

*Traits include CBR (callus browning rate), CDR (callus differentiating rate), CPN (callus plantlet number), CRR (callus rooting rate), and GCR (green callus rate)*.

a *Method numbers correspond to (1) mrMLM, (2) FASTmrEMMA, (3) ISIS EM-BLASSO, and (4) pLARmEB*.

b *r^2^ (%), phenotypic variation of traits explained by each QTN*.

*The values in parentheses denote the means for QTNs in different environments*.

Remarkably, 10 QTNs were co-detected not only in multi-environment (including environment and the BLUP model) but also by different methods (Table 3). Among these QTNs, the three QTNs (SYNGENTA15901, SYN39155, and SYN32084) were detected by all the methods as well as in BLUP model and CZ (Table 3). Furthermore, two other QTNs (SYN8267 and PZE-107005556) that are associated with CBR and CRR were identified by three methods and in two environments. The remaining six QTNs were associated with CPN, CRR, and GCR, and they were identified by two methods and found in two locations (Table 3).

**Table 3:** Stably expressed QTNs in both multi-environment (including BLUP model) and multi-method.

**Trait**	**Marker**	**Method (1, 2, 3, 4)^a^**	**Environment and BLUP**	**LOD score**
CBR	SYNGENTA15901	1, 2, 3, 4	BLUP and CZ (2)	3.74 and 7.63
	SYN8267	2, 3, 4	BLUP and CZ (3)	3.14 and 5.35
CDR	SYN39155	1, 2, 3, 4	BLUP and CZ (3)	4.59 and 4.76
	SYN32084	1, 2, 3, 4	BLUP and CZ (3)	8.87 and 6.35
CPN	PZE-101220149	1, 3	BLUP and CZ (1)	4.42 and 8.26
	SYN39155	1, 3	BLUP and CZ (1)	3.06 and 3.85
CRR	PZE-107005556	2, 3, 4	BLUP and YJ (3, 4)	3.90 and 6.29, 3.43 and 4.47
	SYN35026	3, 4	BLUP and YJ (3, 4)	4.72 and 3.28, 9.40 and 7.37
GCR	SYNGETA13688	1, 3	BLUP and YJ (1)	4.08 and 4.26
	PZE-104024889	2, 3	BLUP and CZ (2)	3.66 and 3.31
	PZE-102138070	3, 4	CZ and YJ (3)	6.33 and 9.02

*Traits include CBR (callus browning rate), CDR (callus differentiating rate), CPN (callus plantlet number), CRR (callus rooting rate), and GCR (green callus rate)*.

a *Method numbers correspond to (1) mrMLM, (2) FASTmrEMMA, (3) ISIS EM-BLASSO, and (4) pLARmEB*.

*JH, CZ, and YJ denote the population planted in Jinghong (2014), Chongzhou (2015), and Yuanjiang (2015), respectively*.

### Distribution of superior alleles in elite inbred lines

**Figure 5:**
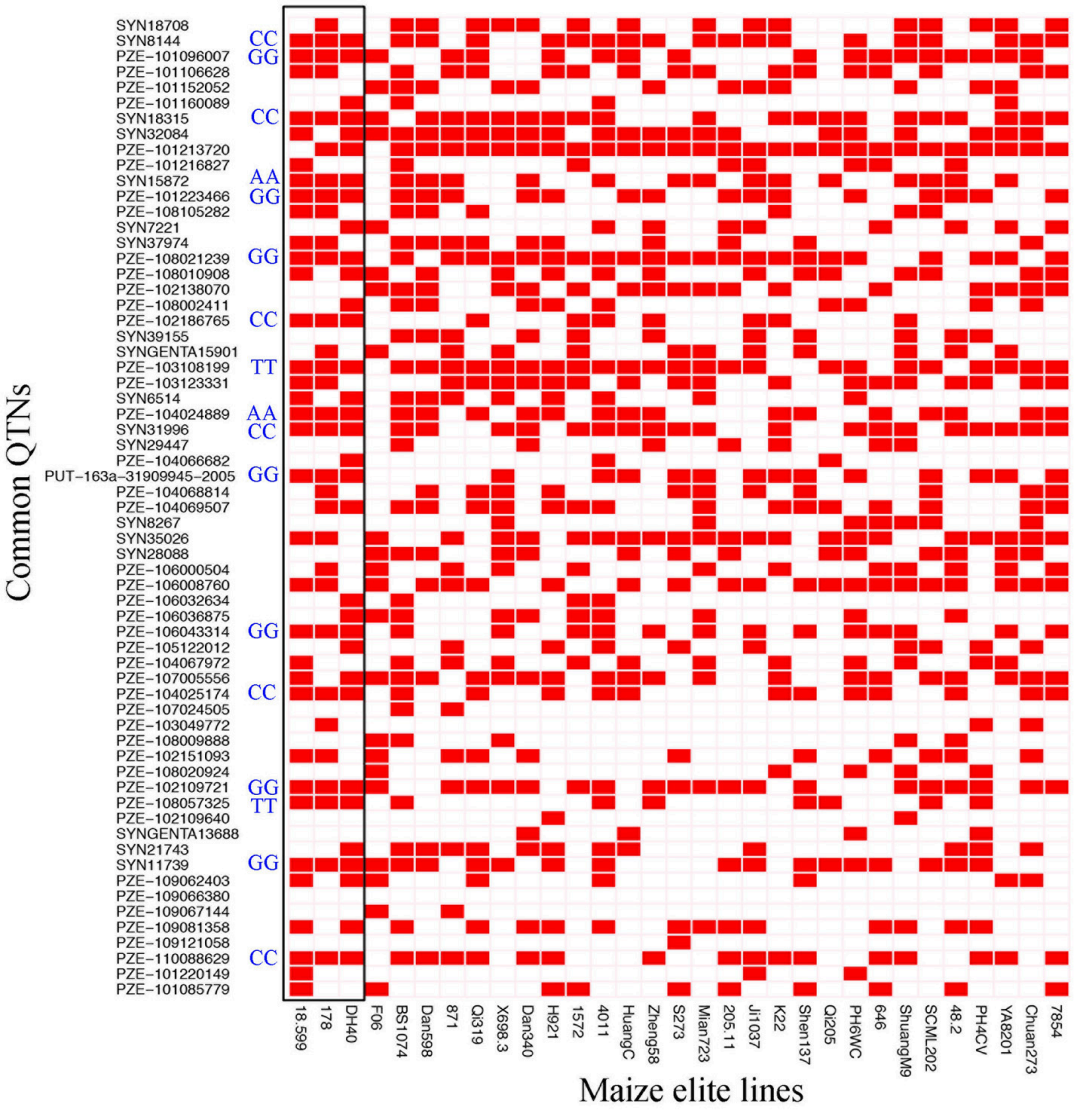
Heat map of the superior alleles distribution for the 63 QTNs in 31 elite inbred lines. Red and White colors represent superior and inferior alleles, respectively. Black box means the superior alleles distribution for the 63 QTNs in high-regeneration lines 18-599, 178, and DH40. AA, TT, GG, and CC represent the genotypes for common superior alleles in 18-599, 178, and DH40.

The 63 common QTNs, detected in multiple environments or using multiple methods, were considered as important QTNs associated with regenerative capacity-related traits. Since 31 elite inbred lines were included in the constructed panel, this enabled us to evaluate the utilization of superior alleles during maize breeding. Herein, the allele associated with a higher phenotypic value was defined as the superior allele for each of the traits, except for CBR and CRR, because callus browning and callus rooting are both disadvantageous phenotypes for regeneration. As described in Table 4, the superior allele percentages for the QTNs ranged from 0.00 to 96.67% in the elite lines, with 27 of the QTNs containing ≥50% superior alleles while the remaining 36 QTNs contained <50% (Figure 5; Table 4). Three QTNs (PZE-101213720, PZE-103108199, and PZE-108021239) had superior allele percentages >80%, while eight (PZE-104066682, PZE-103049772, PZE-101220149, PZE-107024505, PZE-102109640, PZE-109067144, PZE-109121058, and PZE-109066380) had percentages <10% (Figure 5; Table 4).

**Table 4:** Distribution of superior alleles in stably expressed QTNs among 31elite inbred lines.

**QTN**	**Superior alleles**	**Percentage (%)^a^**	**QTN**	**Superior alleles**	**Percentage (%)^a^**	**QTN**	**Superior alleles**	**Percentage (%)^a^**
PZE-101213720	GG	96.67	SYN15872	AA	54.84	PZE-108002411	AA	32.26
PZE-103108199	TT	83.87	PUT-163a-31909945-2005	GG	53.33	PZE-102186765	CC	32.26
PZE-108021239	GG	80.65	PZE-106043314	GG	53.33	PZE-106036875	CC	32.26
SYN18315	CC	76.67	SYN18708	TT	51.61	PZE-101216827	CC	26.67
SYN31996	CC	75.86	PZE-108010908	CC	50.00	PZE-108105282	CC	25.81
SYN35026	AA	74.19	PZE-104025174	CC	50.00	PZE-109062403	AA	25.81
SYN32084	AA	73.33	PZE-109081358	CC	48.39	SYN29447	AA	24.14
PZE-102109721	GG	73.33	SYN28088	CC	46.67	SYN8267	AA	24.14
SYN8144	CC	70.00	SYN21743	TT	43.33	PZE-108009888	GG	19.23
PZE-106008760	GG	67.74	SYN37974	CC	42.86	PZE-108020924	CC	16.67
PZE-107005556	AA	67.74	PZE-104067972	CC	40.00	PZE-106032634	GG	13.33
PZE-110088629	CC	65.52	PZE-102151093	GG	40.00	PZE-101160089	TT	12.90
SYN39155	GG	64.52	PZE-101152052	AA	38.71	SYNGENTA13688	AA	12.90
PZE-101096007	GG	63.33	PZE-106000504	AA	38.71	PZE-104066682	GG	9.68
PZE-103123331	AA	63.33	SYNGENTA15901	CC	38.71	PZE-103049772	GG	9.68
SYN11739	GG	61.29	PZE-104068814	AA	38.71	PZE-101220149	GG	9.68
PZE-101223466	GG	60.00	PZE-108057325	TT	35.71	PZE-107024505	TT	7.14
PZE-104024889	AA	60.00	PZE-101085779	CC	35.71	PZE-102109640	AA	6.45
PZE-101106628	TT	58.62	SYN6514	GG	33.33	PZE-109067144	GG	6.45
PZE-102138070	TT	58.62	PZE-105122012	CC	33.33	PZE-109121058	CC	3.23
PZE-104069507	GG	56.67	SYN7221	AA	32.26	PZE-109066380	TT	0.00

a *Percentage (%) was calculated as: (superior allele number within the 31 elite inbred lines/total allele number for the 31 elite inbred lines) × 100%*.

Moreover, 18 of the elite lines that contained 26–40 superior alleles showed higher phenotypic values, with increased percentages of 109.81% (CDR), 32.91% (CPN), and 75.63% (GCR), relative to the other 13 elite lines that contained 10–25 superior alleles (Table 5 and Table S6). However, for CBR and CRR, the 18 elite lines that contained between 26 and 40 superior alleles had the averaged phenotypic values of 34.17 and 9.68, respectively, which were 26.08 and 28.55% lower than the other 13 elite lines that contained 10–25 superior alleles (Table 5 and Table S6). These findings suggest that the superior alleles have obviously additive effects on regenerative capacity. Therefore, the maize callus regenerative capacity can be improved by increasing the numbers of superior alleles in the lines with low regenerative capacities by marker assisted selection (MAS). Among them, CDR and GCR are the most attractive traits for MAS modification due to them having the most significant enhancement effect. In addition, we found some lines with high regenerative capacity shared common superior alleles, such as lines 178, 18-599, and DH40 which all contained the superior alleles of SYN15872, PZE-104024889, PZE-103108199, PZE108057325, PZE-101096007, PZE-101223466, PZE-108021239, PUT-163a-31909945-2005, PZE-106043314, PZE-102109721, SYN11739, SYN8144, SYN18315, PZE-102186765, SYN31996, PZE-104025174, and PZE-110088629 (Figure 5). This suggested these superior alleles may play an important role in callus regeneration process. All these findings will be more useful in the application of superior alleles in maize breeding.

**Table 5:** Phenotypic values of different numbers of superior alleles for the five traits among the common QTNs within the 31 elite lines.

**Trait**	**Mean phenotypic value in three environments**	**Mean phenotypic value in three environments**	**Increased percentage (%)**
	**(contain 10–25 superior alleles)**	**(contain 26–40 superior alleles)**	
CBR	46.22	34.17	−26.08
CDR	11.30	23.70	109.81
CPN	1.09	1.45	32.91
CRR	13.54	9.68	−28.55
GCR	27.14	47.66	75.63

*Traits include CBR (callus browning rate), CDR (callus differentiating rate), CPN (callus plantlet number), CRR (callus rooting rate), and GCR (green callus rate)*.

### Candidate genes determined based on common QTNs

According to the 63 common QTNs, we further focused on the associated candidates. The results showed that a total of 40 candidate genes were obtained based on the B73 genome (RefGen_v2, Table 6). Among them, 8, 11, 7, 8, and 13 candidate genes were associated with CBR, CDR, CPN, CRR, and GCR, respectively (Table 6). Moreover, one QTN correlated with the CDR trait was associated with GRMZM2G589579 and had the largest LOD-value of 16.25 (Tables 2, 6). Based on the functional annotations, these genes were mainly classified as transcription factors and kinases (Table 6). Specifically, seven genes were located on chromosomes 1, 2, 3, and 6, with each associated with two of the regeneration capacity-related traits (Table 6). In detail, gene models GRMZM2G108933, GRMZM2G072264, and GRMZM2G026095 were individually correlated with both CBR and GCR, while GRMZM2G309660 was associated with CBR and CRR (Table 6). Moreover, GRMZM2G163761 was correlated with CDR and GCR, while GRMZM2G097959 and GRMZM5G835276 were associated with CDR and CPN (Table 6).

**Table 6:** Candidate genes based on the stable commonly expressed QTNs.

**Trait**	**Marker**	**Chromosome**	**Position (bp)**	**Candidate genes (v2)**	**Annotation**
CBR GCR	PZE-103123331	3	181,066,730	GRMZM2G108933	(*WOX*2) WUSCHEL related homeobox 2
CBR GCR	SYN7221	2	6,200,684	GRMZM2G072264	RNA-binding (RRM/RBD/RNP motifs) family protein
GCR CBR	PZE-102138070	2	186,820,524	GRMZM2G026095	Tuliposide A-converting enzyme 1, chloroplastic
CBR CRR	SYN6514	3	196,351,287	GRMZM2G309660	Unknown
CDR GCR	SYN32084	1	256,515,262	GRMZM2G163761	kip1 (knotted interacting protein1)
CPN CDR	SYN39155	3	2,446,145	GRMZM2G097959	GTP binding
CDR CPN	PZE-106032634	6	75,630,749	GRMZM5G835276	Alpha-L-fucosidase 2
CBR	SYNGENTA15901	7	5,038,808	GRMZM2G060866	Anther-specific proline-rich protein APG
CBR	PZE-101213720	1	264,163,677	GRMZM2G123204	Adenylosuccinate synthetase, chloroplastic
CBR	PZE-101152052	1	195,484,495	GRMZM2G138425	Hypothetical protein
CBR	SYN8267	4	169,213,008	GRMZM2G383210	jmj21—JUMONJI-transcription factor 21
CDR	SYN15872	8	161,523,427	GRMZM2G371033	sbp18 (SBP-transcription factor 18)
CDR	PZE-101216827	1	267,908,158	GRMZM2G066749	dek35 (defective kernel35)
CDR	SYN31996	6	163,506,361	GRMZM2G136219	Unknown
CDR	PZE-108002411	8	2,512,300	GRMZM2G589579	ago4a (argonaute4a)
CDR	PZE-101096007	1	94,367,481	GRMZM2G088524	mybr32 (MYB-related-transcription factor 32)
CDR	PZE-106036875	6	84,672,851	GRMZM2G088930	Midasin
CDR	PUT-163a-31909945-2005	6	110,706,817	GRMZM2G412611	Alpha-glucan water dikinase 1 chloroplastic
CDR	SYN8144	10	142,358,869	GRMZM2G033724	Trypsin family protein
CPN	PZE-108105282	8	159,954,599	GRMZM2G460576	Unknown
CPN	PZE-104066682	4	131,771,972	GRMZM2G122983	Vacuolar protein sorting-associated protein 20 homolog 2
CPN	PZE-106043314	6	93,212,668	GRMZM2G047969	Protein JASON
CPN	PZE-102186765	2	230,884,488	GRMZM2G082302	Unknown
CPN	SYN2944	5	213,294,101	GRMZM2G017868	Unknown
CRR	PZE-101160089	1	202,300,686	GRMZM2G315375	br2 (brachytic2)
CRR	SYN35026	5	1,946,471	GRMZM2G415491	rh3 (RNA helicase3)
CRR	SYN18315	1	252,377,691	GRMZM2G165042	bhlh43 (bHLH-transcription factor 43)
CRR	PZE-106008760	6	25,291,385	GRMZM2G168441	Putative HLH DNA-binding domain superfamily protein
CRR	SYN28088	5	68,653,887	GRMZM2G168603	MDIS1-interacting receptor like kinase 1
CRR	PZE-106000504	6	1,234,387	GRMZM2G137894	Pentatricopeptide repeat-containing protein At2g33760
CRR	SYN18708	1	21,466,619	GRMZM2G004397	pco148373a Syntaxin/t-SNARE family protein
GCR	PZE-104024889	4	28,985,737	GRMZM2G130442	ocl5a (outer cell layer5a)
GCR	PZE-104068814	4	136,958,099	GRMZM2G034697	Phosphatidyl-N-methylethanolamine N-methyltransferase
GCR	PZE-108010908	8	11,504,308	GRMZM2G112968	Unknown
GCR	SYN37974	2	10,782,867	GRMZM2G068982	Methionine aminopeptidase
GCR	PZE-103108199	3	169,053,554	GRMZM2G028252	Hypothetical protein
GCR	PZE-104069507	4	138,153,696	GRMZM2G133226	Nucleotide/sugar transporter family protein
GCR	PZE-101106628	1	110,914,630	GRMZM2G368632	Cysteine-rich receptor-like protein kinase 10
GCR	PZE-101223466	1	274,722,612	GRMZM2G001869	Unknown
GCR	PZE-108021239	8	20,231,393	GRMZM2G168933	Hypothetical protein

*The traits include CBR (callus browning rate), CDR (callus differentiating rate), CPN (callus plantlet number), CRR (callus rooting rate), and GCR (green callus rate)*.

### Expression patterns of candidate genes

**Figure 6:**
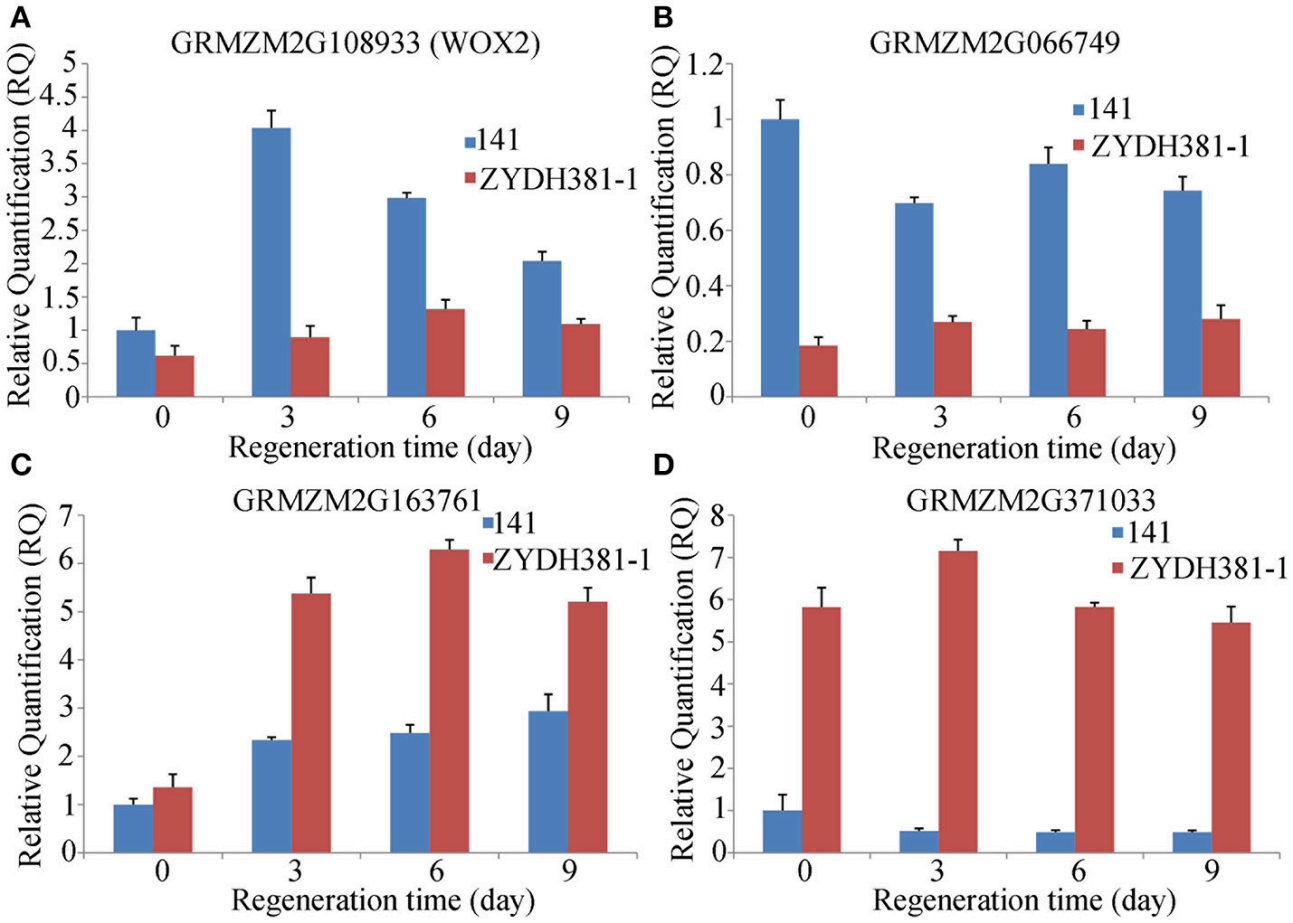
Expression levels of candidate genes at different regeneration stages. Here, 141 is a line with high regeneration and ZYDH381-1 IS the one with low regeneration. **(A–D)** Represents the relative expression levels of GRMZM2G108933 (WOX2), GRMZM2G066749, GRMZM2G163761 and GRMZM2G371033, respectively.

To detect the responses of the candidate genes to callus regeneration, two lines 141 (with high regenerative capacity) and ZYDH381-1 (with low regenerative capacity) were submitted to qRT-PCR analysis for four randomly selected genes at three regenerative stages (3 d, 6 d, and 9 d) and CK (0 d). Among these genes, *WOX2* was up-regulated at all of the stages compared to 0 d in 141 and ZYDH381-1. However, the expression level of *WOX2* in line 141 was higher than that in ZYDH381-1 at each of the stages. Besides, the expression peak occurred at 3 d in 141, which was at 6 d in ZYDH381. These indicate that *WOX*2 was more susceptive in the response of callus regeneration in 141 (Figure 6A). GRMZM2G066749 was down-regulated at every of regenerative stage when compared with 0 d in 141, whereas it was slightly up-regulated in ZYDH381-1. Interestingly, the expression level of GRMZM2G066749 in 141 was much higher than that in ZYDH381-1 at all of the stages including 0 d (Figure 6B). However, the expression levels of GRMZM2G163761 and GRMZM2G371033 were generally higher in ZYDH381-1 than those in 141 (Figures 6C,D). These findings suggested that the difference of expression patterns in different lines could be an important factor which influenced the regenerative capacity of embryonic callus.

## Discussion

### Population selection

A population of 144 inbred lines was used for the present study, which is slightly smaller than in other maize GWAS studies (Pace et al., 2014, 2015; Zhang et al., 2016). This is due to the specialty of these maize callus regenerative ability-related traits, which are based on the embryonic callus induction. In our previous study, 362 inbred lines were utilized to identify embryonic callus induction, and only 144 lines had a relatively efficient induction. Therefore, the present study is based on a comparatively small maize population. Moreover, population structure analysis showed that this novel population was divided into two subpopulations. The average LD decay distance was 220 kb (*r*^2^ = 0.2), which was relatively consistent with the distance obtained for the initial population of 362 inbred lines (Zhang et al., 2016). This finding indicates that the average LD decay distance is almost stable despite the reduced population size. Additionally, some QTNs for the five traits were co-identified in different methods and multi-environment (in Results section). Of particular interest is the candidate gene *WOX2* (in Candidate Gene Functions in Callus Regenerative Capacity section), which has been proven to promote the formation of resistant seedlings after callus transformation. These findings confirm the reasonability of the population structure used for this study.

### Advantages of the new multi-locus GWAS approaches

Previous studies have dissected some complex traits using a GLM or MLM based on a single-locus GWAS (Zhang et al., 2005; Yu et al., 2006; Pace et al., 2015). However, both of these two models have procedural limitations. GLM has a high false positive rate (FPR) because this model does not correct the population structure (Q) or polygenic background (K; Korte and Farlow, 2013). In the MLM, the correction of Q and K is so stringent that many significant loci are missed, especially small-effect loci (Wang et al., 2016). In recent years, researchers developed some multi-locus methodologies to address these limitations, such as mrMLM, FASTmrEMMA, ISIS EM-BLASSO, and pLARmEB (Wang et al., 2016; Tamba et al., 2017; Wen et al., 2017; Zhang et al., 2017a), and they were used in this study. In these new multi-locus methods, the significance level was set to a LOD score = 3, which was equivalent to *P* = 0.0002 (Wang et al., 2016). However, in the single-locus MLM GWAS methods, the significance threshold is generally set to *P* = 0.05/*m* (*m* is the number of markers), thus the multi-locus GWAS methods are less stringent. Furthermore, FPRs for these four multi-locus GWAS approaches were smaller than in the single-locus MLM GWAS methods and other multi-locus GWAS methods (Wang et al., 2016; Tamba et al., 2017; Wen et al., 2017; Zhang et al., 2017a). Therefore, these methods were considered effective alternative approaches (Wang et al., 2016; Tamba et al., 2017; Wen et al., 2017; Zhang et al., 2017a). In this study, 127, 56, 160, and 130 significant QTNs were found for the five traits using mrMLM, FASTmrEMMA, ISIS EM-BLASSO, and pLARmEB, respectively (Figure 3; Tables S2–S5). However, only one and six significantly associated SNPs were detected when using MLM (R package GAPIT) and FarmCPU (R package FarmCPU; PCA+K, where PCA and K were calculated by GAPIT and SpAGeDi, respectively) models, respectively (*P* = 0.05/43427 = 1.15 × 10^−6^; Table S7). This suggested that these multi-locus methods were more powerful when used for detecting the QTNs for regeneration-related traits of maize. Furthermore, some stably expressed QTNs were commonly detected in multiple environments (or between environment and the BLUP model) (Table 1) and a total of 58 common QTNs were identified by multiple methods (Table 2). These evidence verified the reliability of these new multi-locus methods. Comparison of the four methods illustrates that ISIS EM-BLASSO is slightly more powerful than the other three methods (Figure 3). Additionally, the running time for these four methods when using the data generated herein are as follows: mrMLM > FASTmrEMMA > pLARmEB > ISIS EM-BLASSO (Figure S5). Notably, the validated functional gene *WOX2* (mentioned above) was commonly detected in multiple methods for both CBR and GCR. These findings suggest that the most robust approach enabling the identification of the most interesting candidate genes is to use a combination of the methods utilized herein.

### Application of superior alleles in maize breeding

When examining the common QTNs within the 31 elite inbred lines, 36 of the 63 QTNs contained <50% superior alleles (Table 4), suggested that these alleles were not effectively selected during artificial selection. A possible reason is that maize regenerative ability was not previously a main breeding focus. Instead, breeding efforts have focused on yield-related traits, plant type-related traits, resistance-related traits, and high quality-related traits. In the remaining 27 common QTNs, superior alleles proportions ≥50% was observed, with three of these QTNs (PZE-101213720, PZE-103108199, and PZE-108021239) having proportions >80% (Table 4). These findings suggest that in some cases, these superior alleles must be linked with traits of interest for breeders and thus were maintained during artificial selection.

The results presented herein show that the identified superior alleles exhibited additive effects on the regenerative capacity. Furthermore, this study focused on the number of superior alleles in several popular inbred lines (Zheng 58, PH4CV, and PH6WC), whose high yields and high combining abilities were outstanding (Barker, 2005; Ma et al., 2014; Li et al., 2017). The results showed that for each line, the superior allele proportion was <50% in the 63 QTNs (Figure 5, Table S6). Future studies could focus on these lines acquiring more super alleles and an improved regenerative ability that will contribute to the establishment of callus regeneration and a transformation system. These findings also enable the furthering of gene functional research in these lines.

We further investigated the distribution of these superior alleles in those lines that failed to induce the callus. As a result, the proportions of the superior alleles in different lines ranged from 20.64 to 57.14% and the average value was 40.27%, which was very similar to the averaged proportion (40.96%) of superior alleles in the 144 inducible lines. In addition, the averaged proportion of superior alleles for *WOX2* (PZE-103123331) was 69.29 and 70.14%, respectively, in the uninduciable and induciable lines (these data were not provided). These suggested that these QTNs associated with callus regeneration were probably not involved in the induction of embryonic callus.

### Candidate genes involved in callus regenerative capacity

Based on the identified common QTNs, 40 candidate genes were identified, with several previously reported to be associated with transgenic callus regeneration, auxin transport, cell fate, seed germination, or embryo development (Table 6). These gene included GRMZM2G108933, GRMZM2G130442, GRMZM2G315375, GRMZM2G163761, GRMZM2G412611, GRMZM2G066749, and GRMZM2G371033. GRMZM2G108933, which was associated with CBR and CDR, was annotated to *WOX2*, an embryonic transcription factor (Nardmann et al., 2007) (Table 6). In *Arabidopsis, WOX5* is closely associated with the root stem cell niche (Sarkar et al., 2007). In the recent year, *WOX2* (a homologous gene to GRMZM2G108933) was introduced into maize by genetic transformation, and it increased the rate of resistant seedlings from transformed immature embryos (Lowe et al., 2016). These findings suggest that GRMZM2G108933 could be an important functional gene controlling maize callus regeneration by inhibiting callus browning and promoting callus differentiation. GRMZM2G130442 (associated with GCR) and GRMZM2G315375 (associated with CRR) are thought to regulate plant embryo development, which is consistent with their assigned associations herein (Table 6). As a member of the HD-Zip (homeo domain-leucine zipper) family, GRMZM2G130442 was annotated to the *Zea mays* outer cell layer (*ZmOCL*) family (Table 6), which has been reported to play roles in defining different regions of the epidermis during embryonic development and it controls the maintenance of cell-layer identity in meristematic regions (Ingram et al., 2000). GRMZM2G315375, known as *br2*, encodes P-glycoproteins (PGPs) (Table 6), which has been implicated in auxin transport. Meanwhile, auxin is widely accepted to be one of the most important hormones for embryo dedifferentiation (Pasternak et al., 2002). Moreover, Cassani et al. (2011) proposed that the interaction between *br 2* and *br 3* results in an alteration in embryo development. Regeneration is a process involving callus re-differentiation and it is similar to embryo development, but the opposite of embryo dedifferentiation (Yang et al., 2012). Therefore, these findings suggest that GRMZM2G130442 and GRMZM2G315375 could be modulators of callus regeneration.

Gene model GRMZM2G163761 was annotated to KIP1 (knotted interacting protein1) and was associated with CDR and GCR (Table 6). Smith et al. (2002) reported that cell fate in the shoot apical meristem is influenced by the transcriptional regulation from the association of KIP and KN1 (knotted 1), a three amino acid loop extension (TALE) class of homeodomain. Another candidate gene, GRMZM2G412611, which was correlated with CDR was annotated as an alpha-glucan water dikinase 1, chloroplastic-like (Table 6). In wheat, the suppression of glucan water dikinase in the endosperm altered the wheat grain properties, germination, and coleoptile growth (Bowerman et al., 2016). The CDR-associated gene, GRMZM2G066749, was annotated to *dek 35* (defective kernel 35) (Table 6). Clark and Sheridan (1988) demonstrated that *dek 35* is pleiotropic when affecting endosperm, gametophyte, or embryo development by using two non-allelic defective-kernel mutants of maize. These findings indicate that the above genes probably control the callus regenerative capacity by affecting cell fate determination or development of somatic embryo.

## Conclusions

In this study, four new multi-locus GWAS methods were used to identify traits related to regenerative capacity. A total of 127, 56, 160, and 130 significant QTNs, respectively, were identified in mrMLM, FASTmrEMMA, ISIS EM-BLASSO, and pLARmEB for five traits across three environments and the BLUP model. Among these QTNs, 63 were commonly detected in multiple environments or using multiple methods. In total, 40 candidate genes were obtained based on the common QTNs, with several previously reported to correlate with transgenic callus regeneration, auxin transport, or embryo development. For the common QTNs, the percentages of superior alleles ranged from 0.00 to 96.67% within the 31 elite inbred lines. Further analysis revealed that these superior alleles exhibit an additive effect on the regenerative capability of the related traits. These findings suggest that an improvement of the maize callus regenerative ability can be achieved by integrating more superior alleles into maize lines by MAS.

## Author contributions

YS and GP: Designed the experiments; LM, ML, XZ, YY, CQ, YZ, YL, LW, LP, CZ, ZL, YW, and HP: Conducted the experiments and performed the analysis. LM, ML, and YS: Drafted the manuscript. All of the authors provided final approval of this manuscript.

### Conflict of interest statement

The authors declare that the research was conducted in the absence of any commercial or financial relationships that could be construed as a potential conflict of interest.

## Supplementary material

Figure S1 Distribution of 43,427 SNPs on 10 chromosomes.

Figure S2 Predictions of the five traits in three environments by BLUP (Zhang et al., 2017b). The traits include CBR (callus browning rate), CDR (callus differentiating rate), CPN (callus plantlet number), CRR (callus rooting rate), and GCR (green callus rate). CZ, JH, and YJ denote the population planted in Chongzhou (2015), Jinghong (2014), and Yuanjiang (2015), respectively. *R*^2^ denotes the correlation coefficient between the phenotype value mean and corresponding BLUP-value.

Figure S3 Linkage disequilibrium decay in the mapping population. A cutoff of *r*^2^ = 0.2 was utilized.

Figure S4 Manhattan plot of multi-locus GWAS for the five traits. The plots show all of the significant QTNs (LOD score > 3) across three environments and the BLUP model for the four methods (mrMLM, FASTmrEMMA, ISIS EM-BLASSO, and pLARmEB). CZ, JH, and YJ denote the population planted in Chongzhou (2015), Jinghong (2014), and Yuanjiang (2015), respectively. Panels **(A–E)** denotes the significant QTNs for CBR (callus browning rate), CDR (callus differentiating rate), CPN (callus plantlet number), CRR (callus rooting rate), and GCR (green callus rate), respectively.

Figure S5 Running times for the four methods using 43,427 SNPs.

Table S1 Inbred lines and population Q matrix.

Table S2 Significant QTNs for the five traits across three environments and the BLUP model using the mrMLM method. The traits include CBR (callus browning rate), CDR (callus differentiating rate), CPN (callus plantlet number), CRR (callus rooting rate), and GCR (green callus rate). JH, CZ, and YJ denote the population planted in Jinghong (2014), Chongzhou (2015), and Yuanjiang (2015), respectively. *r*^2^ (%), phenotypic variation of traits explained by each QTN.

Table S3 Significant QTNs for the five traits across three environments and the BLUP model using the FASTmrEMMA method. The traits include CBR (callus browning rate), CDR (callus differentiating rate), CPN (callus plantlet number), CRR (callus rooting rate), and GCR (green callus rate). JH, CZ, and YJ denote the population planted in Jinghong (2014), Chongzhou (2015), and Yuanjiang (2015), respectively. *r*^2^ (%), phenotypic variation of traits explained by each QTN.

Table S4 Significant QTNs for the five traits across three environments and the BLUP model using the ISIS EM-BLASSO method. The traits include CBR (callus browning rate), CDR (callus differentiating rate), CPN (callus plantlet number), CRR (callus rooting rate), and GCR (green callus rate). JH, CZ, and YJ denote the population planted in Jinghong (2014), Chongzhou (2015), and Yuanjiang (2015), respectively. *r*^2^ (%), phenotypic variation of traits explained by each QTN.

Table S5 Significant QTNs for the five traits across three environments and the BLUP model using the pLARmEB method. The traits include CBR (callus browning rate), CDR (callus differentiating rate), CPN (callus plantlet number), CRR (callus rooting rate), and GCR (green callus rate). JH, CZ, and YJ denote the population planted in Jinghong (2014), Chongzhou (2015), and Yuanjiang (2015), respectively. *r*^2^ (%), phenotypic variation of traits explained by each QTN.

Table S6 Phenotypic value and superior allele numbers for the 31 elite lines for each trait. The traits include CBR (callus browning rate), CDR (callus differentiating rate), CPN (callus plantlet number), CRR (callus rooting rate), and GCR (green callus rate).

Table S7 SNPs significantly associated with traits detected by GWAS using statistical models MLM and FarmCPU. The traits include CPN (callus plantlet number) and GCR (green callus rate).
