# Supplementary figures and images for: Genetic Dissection of Maize Embryonic Callus Regenerative Capacity Using Multi-Locus Genome-Wide Association Studies
Source: Front Plant Sci. 2018 Apr 26;9:561. doi: 10.3389/fpls.2018.00561 (PMC5933171; doi:10.3389/fpls.2018.00561)

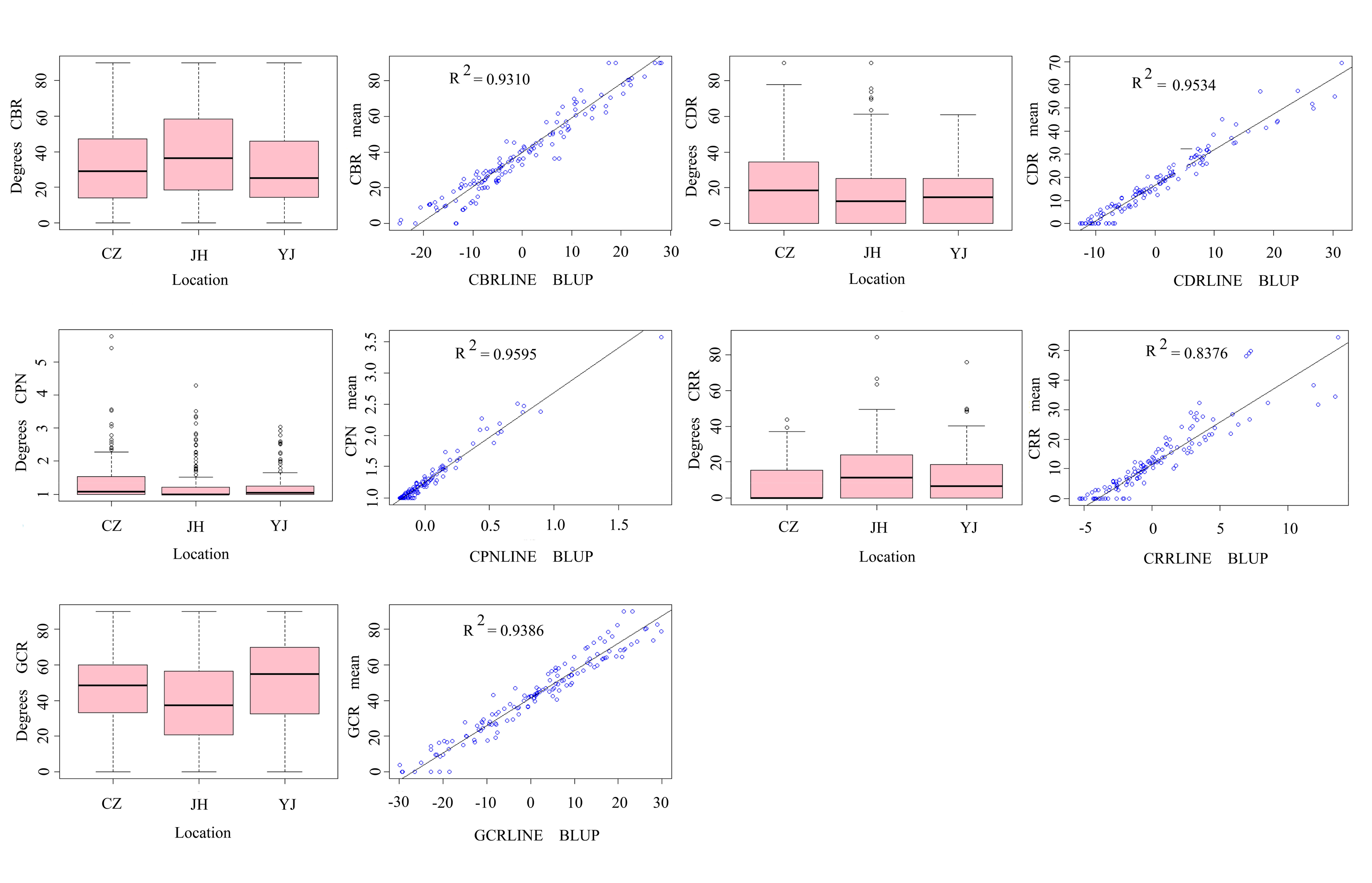

Supplement: Figure S2 — Predictions of the five traits in three environments by BLUP (Zhang et al., 2017b). The traits include CBR (callus browning rate), CDR (callus differentiating rate), CPN (callus plantlet number), CRR (callus rooting rate), and GCR (green callus rate). CZ, JH, and YJ denote the population planted in Chongzhou (2015), Jinghong (2014), and Yuanjiang (2015), respectively. R2 denotes the correlation coefficient between the phenotype value mean and corresponding BLUP-value. [file Image_2.TIF]

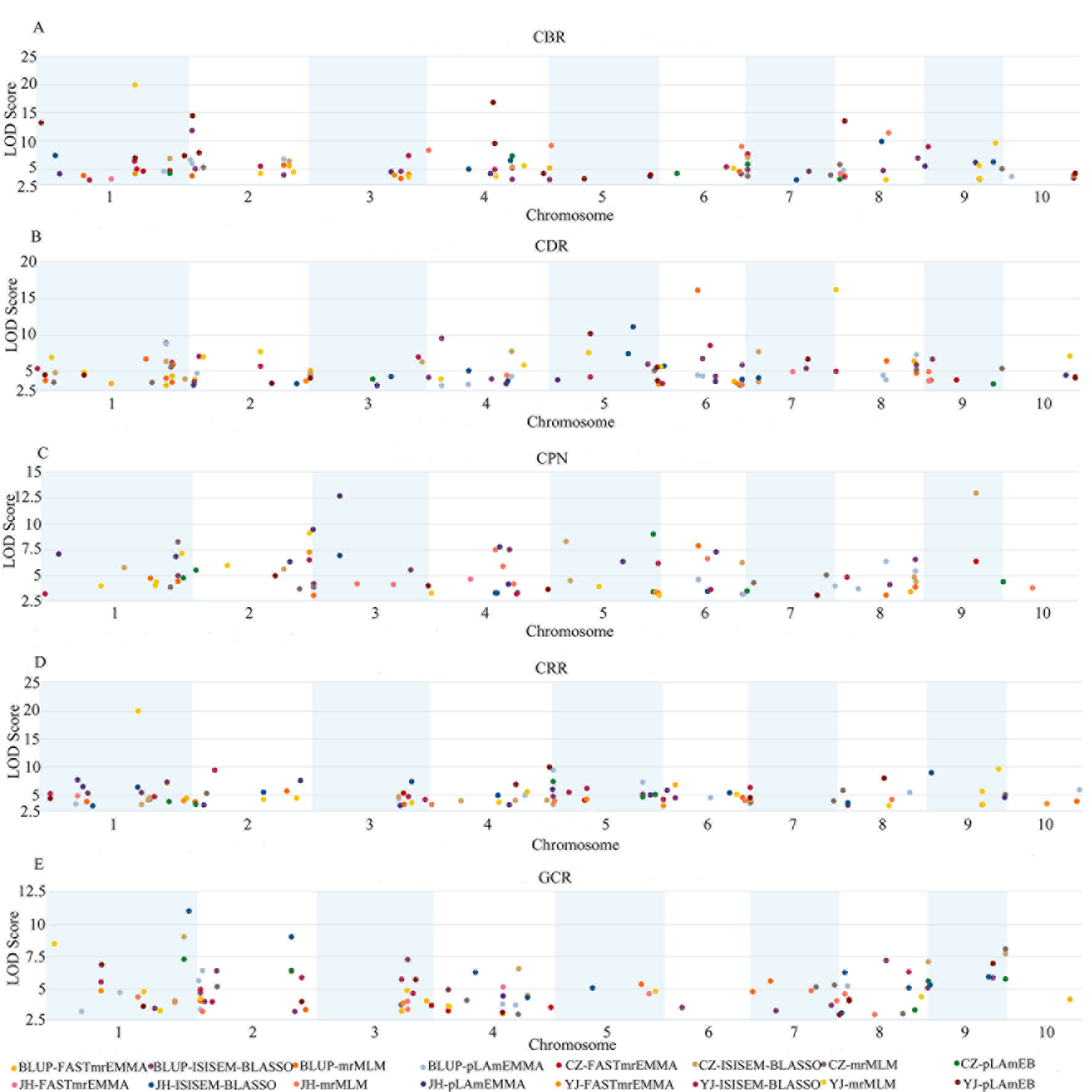

Supplement: Figure S4 — Manhattan plot of multi-locus GWAS for the five traits. The plots show all of the significant QTNs (LOD score > 3) across three environments and the BLUP model for the four methods (mrMLM, FASTmrEMMA, ISIS EM-BLASSO, and pLARmEB). CZ, JH, and YJ denote the population planted in Chongzhou (2015), Jinghong (2014), and Yuanjiang (2015), respectively. Panels (A–E) denotes the significant QTNs for CBR (callus browning rate), CDR (callus differentiating rate), CPN (callus plantlet number), CRR (callus rooting rate), and GCR (green callus rate), respectively. [file Image_4.TIF]
